# Simulation of the Perfusion of Contrast Agent Used in Cardiac Magnetic Resonance: A Step Toward Non-invasive Cardiac Perfusion Quantification

**DOI:** 10.3389/fphys.2019.00177

**Published:** 2019-03-14

**Authors:** João R. Alves, Rafael A. B. de Queiroz, Markus Bär, Rodrigo W. dos Santos

**Affiliations:** ^1^Graduate Program in Computational Modeling, Universidade Federal de Juiz de Fora, Juiz de Fora, Brazil; ^2^Department of Mathematical Modeling and Data Analysis, Physikalisch-Technische Bundesanstalt, Berlin, Germany

**Keywords:** myocardial perfusion imaging, contrast enhanced MRI, porous media, computational physiology and medicine, perfusion quantification

## Abstract

This work presents a new mathematical model to describe cardiac perfusion in the myocardium as acquired by cardiac magnetic resonance (CMR) perfusion exams. The combination of first pass (or contrast-enhanced CMR) and late enhancement CMR is a widely used non-invasive exam that can identify abnormal perfused regions of the heart via the use of a contrast agent (CA). The exam provides important information to the diagnosis, management, and prognosis of ischemia and infarct: perfusion on different regions, the status of microvascular structures, the presence of fibrosis, and the relative volume of extracellular space. This information is obtained by inferring the spatiotemporal dynamics of the contrast in the myocardial tissue from the acquired images. The evaluation of these physiological parameters plays an important role in the assessment of myocardial viability. However, the nature of cardiac physiology poses great challenges in the estimation of these parameters. Briefly, these are currently estimated qualitatively via visual inspection of images and comparison of relative brightness between different regions of the heart. Therefore, there is a great urge for techniques that can help to quantify cardiac perfusion. In this work, we propose a new mathematical model based on multidomain flow in porous media. The model is based on a system of partial differential equations. Darcy's law is used to obtain the pressure and velocity distribution. CA dynamics is described by reaction-diffusion-advection equations in the intravascular space and in the interstitial space. The interaction of fibrosis and the CA is also considered. The new model treats the domains as anisotropic media and imposes a closed loop of intravascular flow, which is necessary to reproduce the recirculation of the CA. The model parameters were adjusted to reproduce clinical data. In addition, the model was used to simulate different scenarios: normal perfusion; endocardial ischemia due to stenosis in a coronary artery in the epicardium; and myocardial infarct. Therefore, the computational model was able to correlate anatomical features, stenosis and the presence of fibrosis, with functional ones, cardiac perfusion. Altogether, the results suggest that the model can support the process of non-invasive cardiac perfusion quantification.

## 1. Introduction

Cardiovascular diseases have a close relation with high blood pressure, smoking, diabetes, lack of exercise, obesity, high blood cholesterol, poor diet, and excessive alcohol (Mendis et al., 2011; Mehta et al., 2015). Many of these conditions can affect *myocardial perfusion* (MP), which is the distribution process of oxygen and nutrients to the cardiac tissue. Among all the injuries related to a compromised cardiac perfusion that can evolve inside the heart, one that occurs frequently is the obstruction of a coronary artery, or stenosis. This leads to perfusion problems, the *coronary artery diseases* (CAD), also referred to as *ischemic heart diseases* (IHD) or simply as *ischemia*. Basically, the stenosis reduces the supply of oxygen and nutrients (perfusion defect) in the region of the myocardium that should be irrigated by the coronary artery in question. This condition may lead to more severe cases, like an infarct, which can provoke irreversible damages to the cardiac tissue, including death. There are several exams used to evaluate myocardial perfusion in clinical practice. Two that are widely used are *computer tomography* (CT) and the *magnetic resonance imaging* (MRI). Both consists in the administration in the patient of a *contrast agent* (CA) which, in the images generated by the chosen protocol, assume a specific contrast on poor perfused regions. The information that can be inferred with the acquired images has a fundamental role in the diagnosis, management, and prognosis of ischemia and infarct. In this work we present a new mathematical model to describe cardiac perfusion in the myocardium as acquired by cardiac magnetic resonance perfusion exams. Our results suggest that the model is an valuable tool that can support the process of non-invasive cardiac perfusion quantification. The next section gives more detailed explanations of MP and of contrast-enhanced MRI, which is the focus of this work.

### 1.1. Background

**Figure 1:**
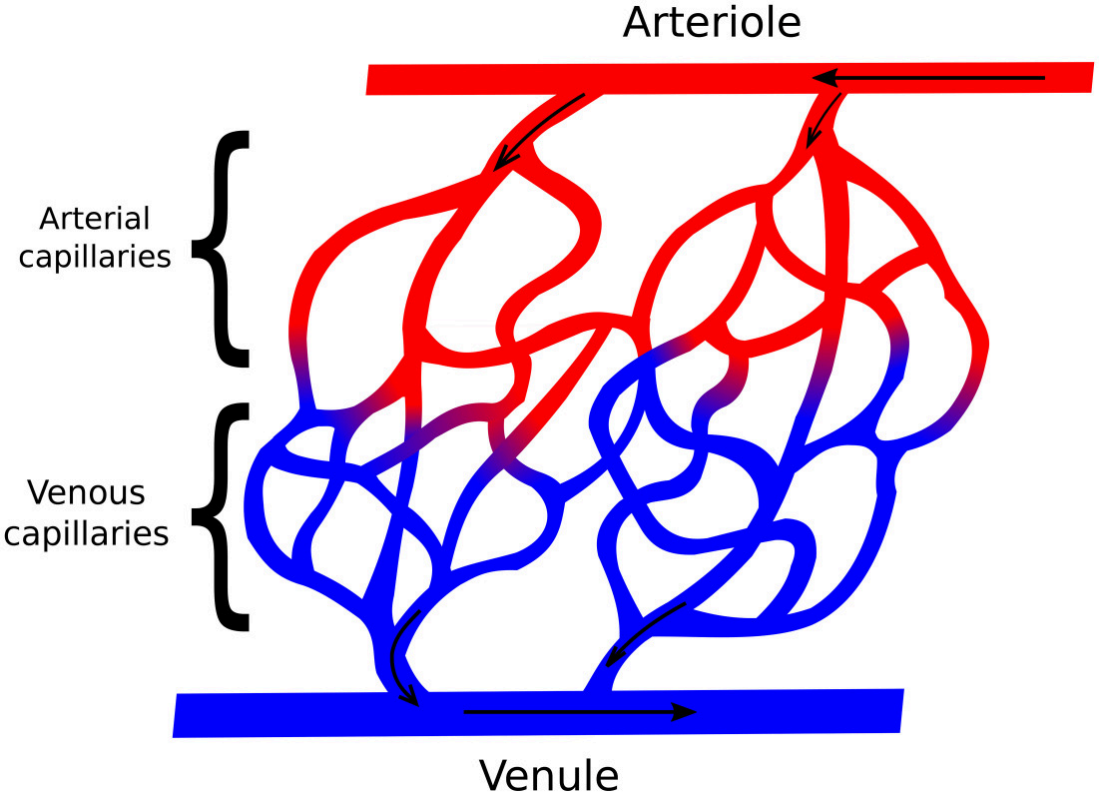
Simplified scheme representing the relation between arteries and veins, in addition with the direction of blood flow. The coronary arteries get smaller and become arterioles, which get smaller again and become arterial capillaries. After the leak of oxygen and nutrients, the arterial capillaries now are called venous capillaries, which get bigger, becoming venules, and finally coronary veins.

For the heart to have the necessary strength to pump blood throughout the body, it needs a constant supply of oxygen and nutrients. This process can be summarized as follows: the blood enters into the myocardium through the epicardial coronary arteries. As Figure 1 summarizes, the coronary arteries branches in smaller arteries, the arterioles. These in turn, branches into even smaller vessels. This process continues down to the capillaries, which are the smallest vessels in terms of diameter. It is on this level where the leakage of oxygen and nutrients from intra to extravascular media happens. After that, the capillaries increase in size and become veins. The venous blood follows the opposite direction and arrives at the venules followed by the coronary veins, these last vessels leave the epicardium to the lungs to be filtrated. Previous studies have shown that the blood-tissue exchange on the capillary level may be a complex phenomenon. For instance, in Bassingthwaighte et al. (1989) and Dash and Bassingthwaighte (2006), a multidomain model that accounts for the blood flow in capillary, endothelium, interstitium and parenchymal cells was developed, that includes the fluid exchange between each domain. However, the present work will not take into account such level of details.

In order to evaluate MP, several exams can be used. One that provides high quality images is *computer tomography* (CT). However, CT uses a harmful and allergenic substance as contrast agent (Brenner and Hall, 2007; Kroeker et al., 2011). Another option is contrast-enhanced MRI (Nikolaou et al., 2011). This one consists in the administration of a dose of *Gadolinium with diethylenetriaminepentacetate* (Gd-DTPA) in the patient that helps to identify patterns in the myocardial tissue. Contrast-enhanced MRI has different protocols. We can mention, for example, the *first pass* (FP) (Gerber et al., 2008) and the *late-enhancement* (LE) (Pop et al., 2013). As the name says, the FP uses the first passage of the CA through the circulatory system to generate the images, in which the regions that are well perfused by the CA acquire a characteristic glow, whereas dark regions indicate problems in perfusion. These problems can be related for instance with ischemia or infarct regions. The FP evaluates the myocardium for 50 s. On the other hand, LE enables the differentiation between a ischemic region, potentially still viable, and infarct. This exam evaluates the myocardium 10 min after the CA administration, and opposite to the FP, the infarct area is the one that gets brighter. Therefore, both FP and LE can be used as complementary MRI perfusion exams.

Nevertheless, the evaluation of the images acquired by the exams is a tedious and error-prone process performed by highly specialized physicians. To support and improve this process different tools that seek a more quantitative evaluation of myocardial perfusion are under development. In Chiribiri et al. (2013) and Alves et al. (2017), such process is studied using phantoms specifically designed to simulate contrast-enhanced MRI exams. In Aquaro et al. (2013), flow quantification is approached by calculating the maximum upslope of the myocardial signal. In Jerosch-Herold (2010) and Zarinabad et al. (2012), the quantification is given in terms of signal deconvolution techniques (Fermi deconvolution). In addition, several mathematical models have been developed with this goal. In Michler et al. (2013), a model is presented that captures pressure and velocity profiles of the blood in 3D representation of the heart. In Alves et al. (2016), the authors performed simulations considering regions with low permeability, showing the behavior of the CA in different scenarios. In Cookson et al. (2014), for the purpose of reproducing the exchange of CA between intra and extravascular spaces, the authors take into account a 2-domain formulation. Although clearly substantial progress has been made, there is still room for further improvements.

Another invasive exam that indirectly evaluates perfusion is the *Fractional Flow Reserve* (FFR) (Lee et al., 2017), which gives information related to the severity of a coronary stenosis. It is important to mention that there are softwares that can estimate FFR by combining mathematical models (also PDEs) and images acquired by CT exams. These softwares are available for clinical use (Hlatky et al., 2015).

In this work, we propose a similar tool that also uses mathematical models based on PDEs and images, in this case from contrast-enhanced MRI exams. The model simulates the blood flow in a short-axis slice of the myocardium (cardiac muscle). Simulations of the model provides the dynamics of CA in the initial stage, as captured by *first pass*, and after many minutes of initial CA injection, as captured by *late enhancement*. In order to describe myocardial blood flow, Darcy approach for fluid in porous media is used, coupled to advection-diffusion-reaction equations to evaluate the CA dynamics. Clinical data were used to adjust the parameters of the model. Some specific contributions of this work are: (1) the simulation of CA dynamics in a domain that represents the short axis of the myocardium; (2) the development and test of a mathematical formulation that takes into account three domains (intravascular, extravascular, and fibrosis); (3) a model that contains a closed loop of intravascular flow, which is necessary to reproduce the recirculation of the CA; (4) the model parameters were adjusted to reproduce clinical data; and (5) analysis of the CA dynamics and its quantification for scenarios of normal perfusion, endocardial ischemia, and myocardial infarct.

In section 2 the multi-domain mathematical model is presented. Section 3 describes the numerical methods. Section 4 presents the results obtained with the simulations. Section 5 presents a general discussion of the results and the limitations of the model. Finally, section 5.3 presents the conclusions.

## 2. Multi-Domain Model for the Blood Circulation and the Contrast Dynamics

In this work, the blood flow in the myocardium is considered as a single-phase flow in porous media. Basically, a porous media is a solid filled with empty spaces, which are connecting each other. The fraction of empty spaces in the total sample volume, which is called *porosity*, is given by

(1) $$\phi =\frac{V_{p}}{V_{t}},$$

where *V*_*p*_ is the volume of the pores and *V*_*t*_ is the total volume of the sample. This kind of analysis is widely used in oil extraction (Taber, 1969) and aquifer contamination by organic compounds (Abriola and Pinder, 1985).

In order to better understand the complex phenomenon of myocardial perfusion and, in particular, how MRI images are related to ischemia or infarct, previous works have considered the myocardial arterial tree as a porous media (Bassingthwaighte et al., 1989; Michler et al., 2013; Cookson et al., 2014; Alves et al., 2015, 2016). This approach represents a major simplification. As presented in Figure 1 the vessel network is discrete and involves non-trivial topological features. Therefore, a lot of information is lost when we represent it by a continuous porous media. Nevertheless, this simplified model is still able to provide useful insights about the phenomena of myocardial perfusion. For instance, in Michler et al. (2013), Darcy's law for flow in porous media was used to model the pressure field and blood velocity in the intravascular tree that perfuses the myocardium. In addition, the same work proposes the use of three different compartments, each one dealing with a diameter range of the vascular tree. In addition to this study, in Alves et al. (2015, 2016), a second equation was proposed, this was a diffusion-advection PDE used to describe the CA dynamics in scenarios of both healthy and diseased tissues. For this purpose, a small region in the domain was set with a lower permeability. In Cookson et al. (2014), a third equation was added to capture the CA dynamics in two different domains, intravascular and extravascular. In this work, we will present a new mathematical model that is able to reproduce three important scenarios: perfusion on a healthy tissue, on an ischemic region, and on an infarct. The last two can happen due to a low pressure difference between epicardial and endocardial surfaces, which can be caused by a stenosis in a coronary artery. Compared to the aforementioned previous work, the new model is more realistic:

- It reproduces the Gd-DTPA recirculation: the CA substance is partially washed out after the first pass in the myocardium. However, as can be seen in Lima et al. (1995) and Daly and Kwong (2013), the blood with CA returns to perfuse the heart in cycles. The new arrival of this CA wave implies in a small increase of the SI (Signal Intensity), which is captured by the new model here presented;
- Here, our domain is not a simple rectangle as those used before in Cookson et al. (2014) and Alves et al. (2016). In this work, we use a realist short-axis cut of the cardiac left ventricle. Therefore, the simulation results are closer to the images actually acquired by an MRI exam;
- The CA is confined to the interstitial (extracellular) space, since it is known that it does not to enter into the cardiac cells (intracellular space);
- When the scenario of infarct is simulated, we added a sortion term in the equations to capture how CA is trapped inside the fibrosis network present in the scars (injured portion of the interstitium).

**Figure 2:**
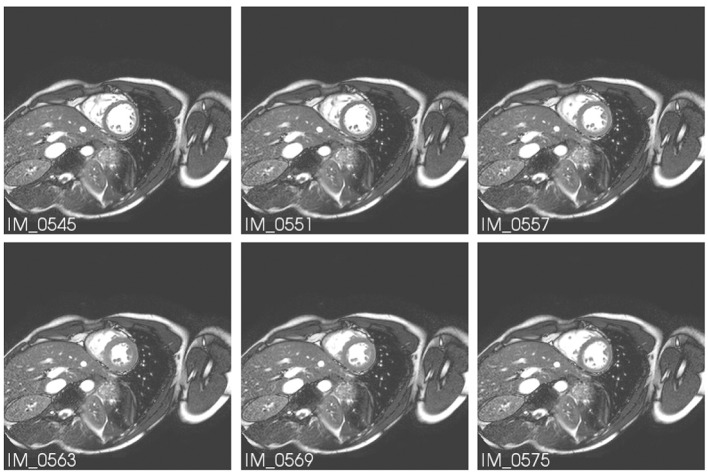
Six MR images at different phases of cardiac cycle (it is possible to observe the movement of the myocardium). Extracted from Koch et al. (2011).

Cardiac perfusion happens together with cardiac contraction, see Figure 2 for an example of the different phases of cardiac contraction as captured by cine MRI exam. However, for contrast-enhanced MRI physicians use a single phase, usually end-diastole, and analyze CA dynamics comparing these images acquired on different heart beats. Therefore, the sequence of contrast-enhanced MR images is analyzed on a non-contracting heart. It is a simplification, but serves well the purpose as most of the perfusion takes place during diastole. In this work, we will use the same hypothesis and consider CA dynamics on a non-contracting heart. Therefore, the variables of interest are myocardial pressure and CA concentration. The blood fluid is taken as incompressible, homogeneous, and Newtonian.

### 2.1. Mathematical Model

**Figure 3:**
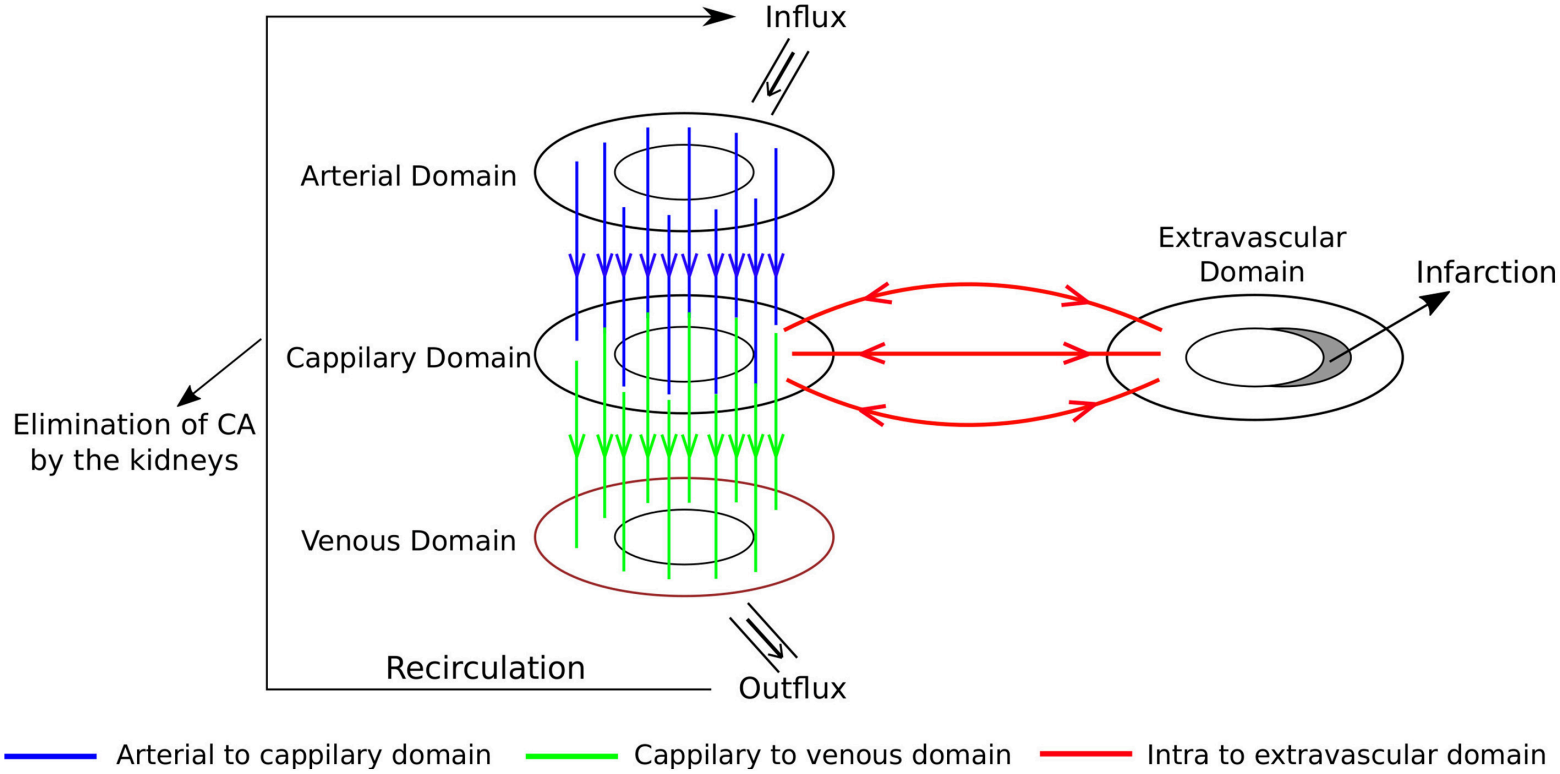
Multidomain model for cardiac perfusion of the left ventricle. Each domain is modeled by a porous media. On the left side of the figure we have the domains that represent the intravascular flow in the heart: arterial domain **(top)**, capillary domain **(middle)**, and venous domain **(bottom)**. On the right side of the figure we have the extravascular domain in the heart which is subdivided in interstitial space and fibrotic network (shaded area associate to an infarction). A recirculation circuit, which is a simplification of the pulmonary and systemic circulation processes, enforces a closed circuit.

Figure 3 shows a framework of a multidomain model for cardiac perfusion of the left ventricle. Each domain is modeled by a porous media where the variables of interest are pressure, flow, and CA concentration. On the left side of the figure we have the domains that represent the intravascular flow in the heart: arterial domain (top), capillary domain (middle), and venous domain (bottom). On the right side of the figure we have the extravascular domain in the heart which is subdivided in interstitial space and fibrotic network (shaded area associate to an infarction). This multidomain model captures the flux between the domains as part of a closed circuit. There is an influx through the coronary arteries on the epicadial surface of the arterial domain. The blood follows to the capillary domain, where there is the exchange of plasma and CA with the extracellular domain. The flow continues from the capillary domain to the venous one, from where the coronary veins leave the heart. This outflux enters a recirculation circuit, which is a simplification of the pulmonary and systemic circulation processes.

**Figure 4:**
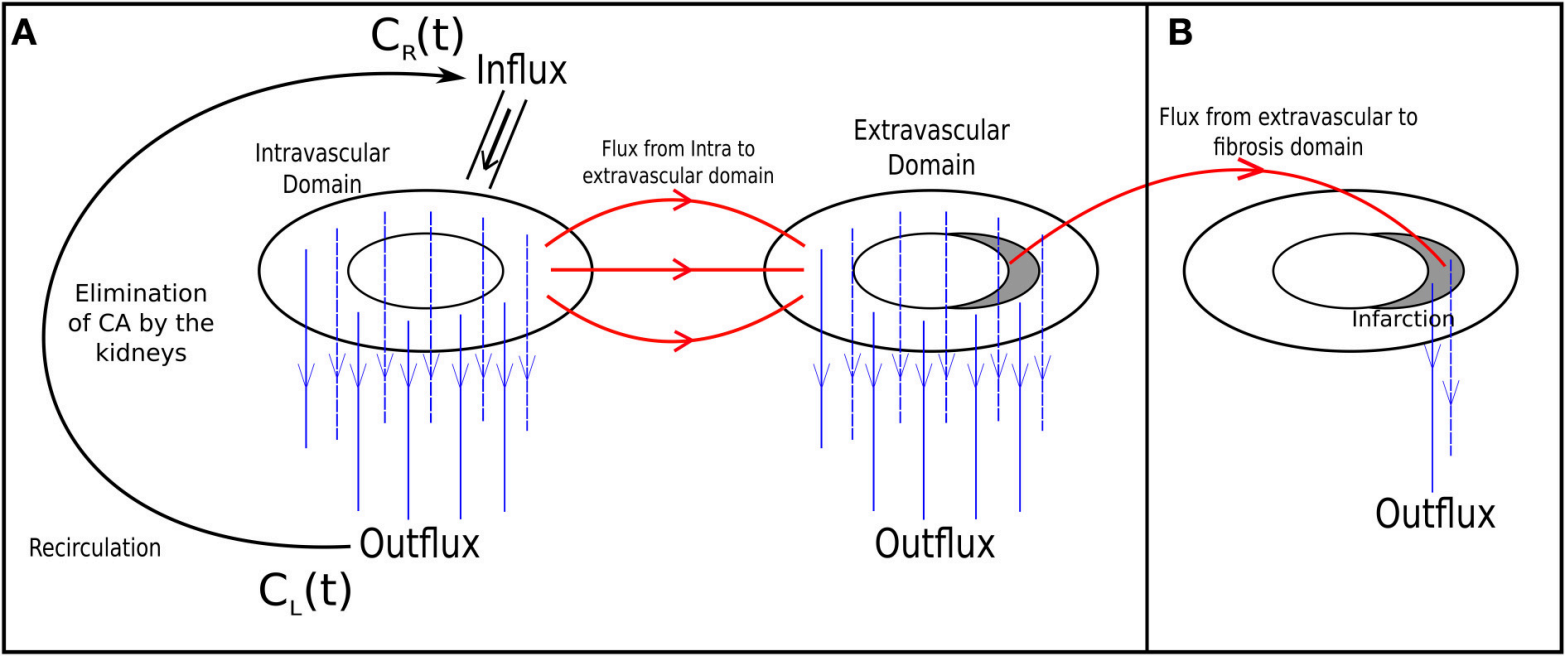
The simplified two-domain model **(A)**, used to simulate healthy and acute ischemia cases; and the three-domain model extended by a fibrosis domain **(B)**, used to simulate an infarct region.

This multidomain model can be further extended or simplified, depending on the main goals of a particular study. For instance, it could be used to reproduce the transmural perfusion and respective pressure gradients from subepicardium to subendocardium in the arterial domain as well as the outward perfusion, i.e., from subendocariaum to subepicardium, in the case of the venous domain. In addition, by setting an appropriate anisotropic permeability tensor of the vascular domain, it may be used to reproduce the preferential perfusion direction of the micro-vascular system, which follows the orientations of cardiac fibers. In this work, we have used a simplified version of the described multidomain approach by combining the arterial and capillary domains into one intravascular domain and by neglecting the venous domain. This simplification is presented in Figure 4A. As our results will show, this simplified model is quite successful in reproducing several aspects of CA dynamics in myocardial perfusion. In particular, it allowed the study of the influence that a coronary obstruction has in the pressure distribution and cardiac perfusion, including scenarios of ischemia and infarction.

To obtain a transmural pressure gradient, boundary conditions are prescribed, which generates a pressure gradient and therefore a blood flow from epi- to endo-cardium. This part of the model can be summarized as follows:

(2) $$\nabla \cdot \vec{v}=0\text{ in }\Omega_{i},$$

(3) $$\vec{v}=-K\nabla p\text{ in }\Omega_{i},$$

where **K** is the permeability tensor; *p* is the pressure; $\vec{v}$ is the Darcy velocity and Ω_*i*_ is the intravascular domain. The boundary conditions for Equation 2, written in terms of pressure, are given by Dirichlet's boundary condition:

(4) $$p=p_{o}\text{ in }{\text{Γ}}_{o},$$

(5) $$p=p_{i}\text{ in }{\text{Γ}}_{i},$$

where Γ_*o*_ and Γ_*i*_ are epicardial and endocardial boundaries, respectively. For the CA dynamics we considered advective ($\vec{v}C$) and diffusive (−*D*∇*C*) components for both the intravascular and extravascular domains. Thus, this system is given by

(6) $$\frac{\partial (\phi C_{i})}{\partial t}+\nabla \cdot \vec{v}C_{i}-\phi \nabla \cdot (D_{i}\nabla C_{i})+f=0\text{ in }\Omega_{i},$$

(7) $$\begin{array}{l} \frac{\partial ((1-\phi )\lambda C_{e})}{\partial t}-(1-\phi )\lambda \nabla \cdot (D_{e}\nabla C_{e})-f+(1-\phi )\lambda k_{e}C_{e}+g\\ =0\text{ in }\Omega_{e}, \end{array}$$

where *f* models the transfer between the two domains and is given by

(8) $$f\left\{ \begin{array}{l} P\times (C_{i}-C_{e}),\;if\;C_{i}>C_{e},\\ 0,\text{ otherwise},\; \end{array}\right.$$

where *P* is the endothelial permeability. This parameter is known to vary for different pathologies (Bassingthwaighte et al., 1989). Section 5 presents the sensitivity of it. ϕ is porosity, i.e., the fraction of intravascular domain, and thus 1−ϕ is the fraction of the extravascular domain. *C*_*i*_ is the concentration of CA in the intravascular domain (Ω_*i*_), *C*_*e*_ is the concentration of CA in the interstitial space (Ω_*e*_). *D*_*i*_ and *D*_*e*_ are diffusion coefficients. −*k*_*e*_*C*_*e*_ models the flux from the interstitial space to the venous system. As mentioned before, the extravascular space can be divided in interstitial (extracellular) and intracellular portions. We assume that the CA does not enter in the myocytes. Thus, λ represents the fraction of extravascular (1−ϕ) space that is due to the interstitium.

As mentioned before, in the case of chronic infarct, scars may develop by the increase of fibrosis to replace dead myocytes. Therefore, when performing the two protocols of *contrast-enhanced MRI perfusion*, two different situations may happen: in *first pass*, because it takes around 50 s, there is not enough time for the CA to reach the fibrotic area. Thus, on this exam, the images provided by the MRI will indicate a dark color in the injured area, whereas the remain will be bright. On the other hand, in *late enhancement*, there is enough time for the CA to reach the area. In addition, the wash out of CA is delayed, since the network of fibrosis will behave as a trap for CA. The described phenomenon, of fluid, particles or substances been attached to a “solid phase,” in physics, is called sortion. Therefore, the dynamics of the CA in the fibrosis is given by

(9) $$\frac{\partial ((1-\phi )\lambda \lambda_{f}C_{f})}{\partial t}+(1-\phi )\lambda \lambda_{f}k_{f}C_{f}-g=0,\text{ in }\Omega_{f}\text{,}$$

where *C*_*f*_ is the concentration of CA in the fibrosis domain (Ω_*f*_) and *k*_*f*_*C*_*f*_ models the flux from the fibrotic network to the venous system. *g* reflects the flow of CA from interstitium to the fibrotic network. It is nonzero only when we simulate the infarct scenario. It is given by

(10) $$g=(1-\phi )\lambda \lambda_{f}k_{ef}C_{e},$$

where *k*_*ef*_ is the rate in which the contrast goes from the interstitium to the fibrosis. λ_*f*_ is the fraction of the interstitium with fibrosis. See Figure 4B.

For the outer boundary condition (Γ_*o*_), a prescribed flux ($\vec{v}Q\left(t\right)$) is used, applying a transient Gaussian formula:

(11) $$Q(t)=\frac{1}{\sigma \sqrt{2\pi }}e^{-\frac{1}{2}\left(\frac{t-t_{peak}}{\sigma }\right)}+X(t,x,y),$$

where σ^2^ reflects the variance of the contrast agent infusion, *t*_*peak*_ is the mean of the Gaussian which sets the time value for the peak of the gadolinium intensity. *X*(*t, x, y*) is a term that must be added to simulate the CA cyclic behavior observed in experiments for the first pass and late enhancement MRI perfusion (Lima et al., 1995; Daly and Kwong, 2013). In the next section we will present more details about this term.

Homogeneous Neumann boundary conditions are used in Γ_*i*_ for both equations:

(12) $$D_{i}\nabla C_{i}\cdot \vec{n}=0\text{ in }{\text{Γ}}_{i},$$

(13) $$D_{e}\nabla C_{e}\cdot \vec{n}=0\text{ in }{\text{Γ}}_{i},$$

where $\vec{\text{n}}$ is the normal vector. The same is assumed for Γ_*o*_ in Equation 7:

(14) $$D_{e}\nabla C_{e}\cdot \vec{n}=0\text{ in }{\text{Γ}}_{o}.$$

As initial conditions, we have:

(15) $$C_{i}(x,0)=C_{i_{0}}(x)\text{ in }\Omega ,$$

(16) $$C_{e}(x,0)=C_{e_{0}}(x)\text{ in }\Omega .$$

### 2.2. 1D Model to Simulate the CA Cyclic Behavior

Like Equation 6, that simulates the contrast agent dynamics in the myocardium, a similar model, however simplified to 1D, was used to capture the recirculation of contrast. Figure 4A uses a line to represent the recirculation. This line connects the outflux and influx via the variables *C*_*L*_(*t*) and *C*_*R*_(*t*), respectively.

Basically, the values *C*_*L*_(*t*) and *C*_*R*_(*t*) are the amount of contrast at the beginning and at the end of the recirculation, respectively. The amount *C*_*L*_(*t*) is given by

(17) $$C_{L}(t)=\frac{\int_{\Omega_{i}}C_{i}\;d\Omega_{i}}{\left|\Omega_{i}\right|}.$$

The value given by Equation 17 represents the average of the contrast in the intravascular medium. It is considered as a Dirichlet condition in the left boundary of the 1D domain. Thus, for a given velocity, the average of contrast travels in the domain through the advection-diffusion-reaction equation

(18) $$\frac{\partial C_{out}}{\partial t}+\nabla \cdot {\vec{v}}_{out}C_{out}-\nabla \cdot (D_{out}\nabla C_{out})+kC_{out}=0\text{ in }\Omega_{out}.$$

The amount obtained in the right boundary of the 1D domain, *C*_*R*_(*t*), is taken as the recirculation term *X*(*t, x, y*) in Equation 11. Physiologically, a portion of the contrast is eliminated in the recirculation, specially by the kidneys. This is modeled by the reaction term *kC*_*out*_. This phenomenon explains the decrease of CA in the subsequent passes in the myocardium. Although the recirculation is more complex and involves the dispersion of the CA in the whole body in different quantities, this simple 1D model captures the CA elimination and its cyclic behavior in the MRI perfusion exam, as presented in section 4.

## 3. Numerical Methods and Experiments

For the discretization, the FVM (Finite Volume Method) formulation was used, which consists in the evaluation of influx and outflux of a control volume around each node of the mesh. The FVM applied to Equation 2 (an elliptic PDE) gives us a linear system with one equation for each node. The control volumes were taken as squares of size equal to *h*. This system was solved by the iterative method of Jacobi (Quarteroni et al., 2010), with an error tolerance (stop criteria) set to 10^−8^. Once the pressure field is known, Equation 3 is used to obtain the velocity in the faces of the control volumes. The velocity is used in the second term of the left side in Equation 6 and the discretization of the diffusive term follows that of the pressure equation. For the diffusive term, the central differences scheme works very well, without any major problems. But for the advective term a more sophisticated scheme must be used to ensure accuracy and convergence of the numerical method. For the numerical approximation of this term, the *third-order polynomial upwind scheme* (TOPUS) was implemented. For more details about this upwind scheme, see Ferreira et al. (2012) and Alves et al. (2016). We used the explicit Euler method in the temporal part of the advection-diffusion-reaction equations. All the mathematical calculations in each step of the discretization can be found in Alves et al. (2016).

### 3.1. Numerical Simulations

**Figure 5:**
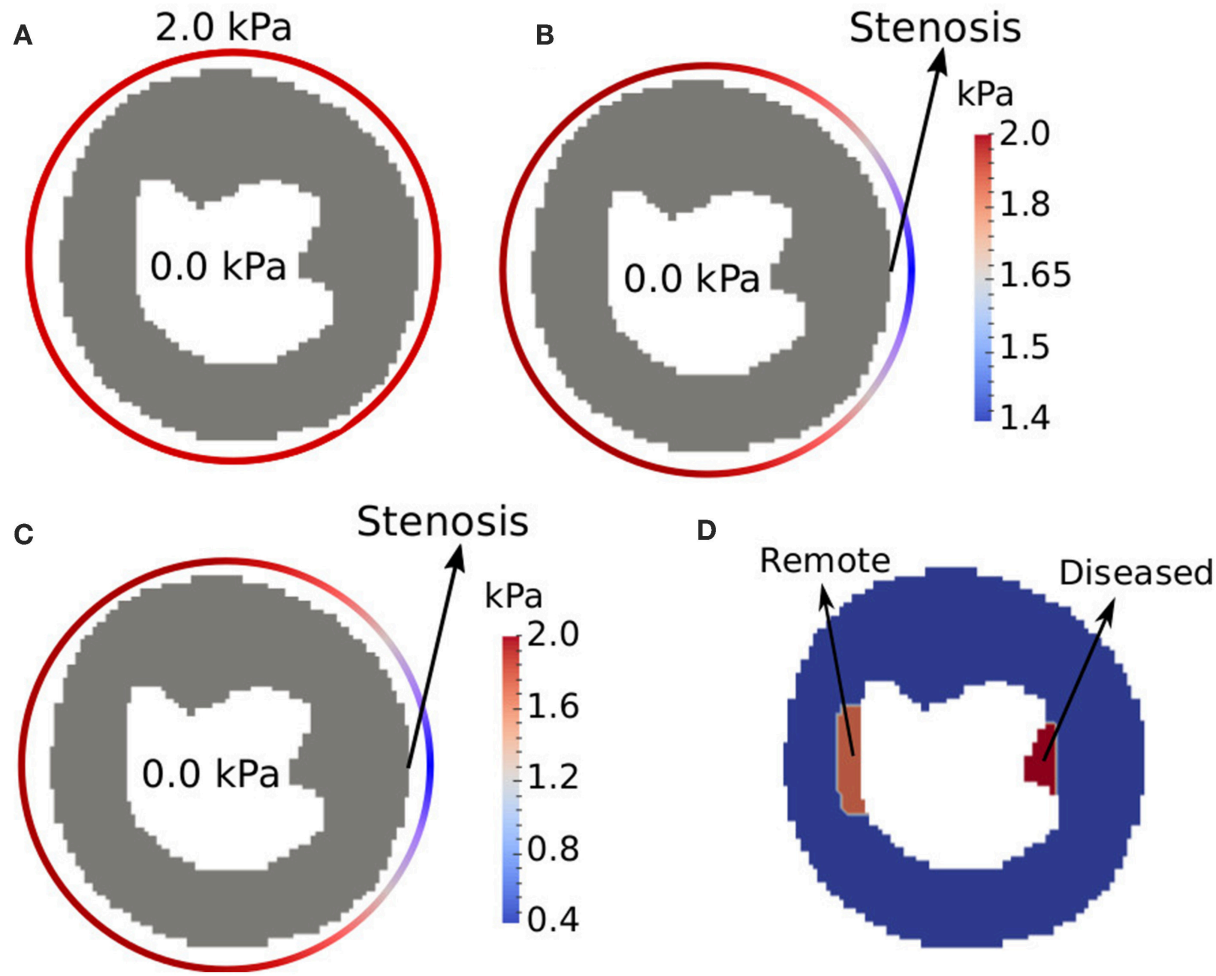
Performed simulations: **(A)** healthy, **(B)** ischemic, and **(C)** infarct. **(D)** Shows the regions of interest for the evaluation of CA signal intensity. When simulating ischemia and infarct, we sum up the concentration of CA in these regions of interest, for each time instant. The colored circle that surrounds the epicardium represents the pressure boundary condition for each scenario. Note the different minimum values of the two color bars.

We simulated the CA dynamics on a short-axis slice of a human ventricle taken from an open repository (Koch et al., 2011) (see Figure 5). During the segmentation process, we have included the papillary muscles as part of the myocardium (Gommans et al., 2016). However, we note that the results presented in this work are not affect by this particular short-axis or segmentation. Similar results can be obtained with different short-axis and different segmentation schemes that do not include the papillary muscles.

We discretized the segmented image using a total of 10,000 finite volumes, i.e., the resolution used for the simulations was Δ*x* = Δ*y* = 0.5mm. The MRI image used had a resolution of 1.5 × 1.5 *mm*^2^ (Koch et al., 2011). Therefore, the resolution used for the simulations was three times higher than the one from the MRI image. However, as we have not processed the segmented image with any smoothing technique, our simulation domain presented in Figure 5 is also pixel-based, with non-smooth boundaries.

In order to assess the model in different scenarios, three main simulations were performed: the first is a healthy situation, with normal values of pressure in epi- and endo-cardium. The second simulation tries to reproduce an ischemia. For this purpose, a drop of pressure is considered in the epicardium, which emulates a coronary stenosis. For the third simulation, we consider a more severe stenosis and a region with fibrosis to reproduce an infarct. The values for the prescribed pressures in the non-ischemic case are taken from Chapelle et al. (2010). For the diseased simulations, the values for the drop of pressure in a location of the epicardium (that simulates a coronary artery) were taken from works that study the FFR technique (Pijls et al., 2000; Sant'Anna et al., 2007; Lee et al., 2017). Here we present the features of each of these scenarios (see Figure 5):

- 1st (normal): *p*(*x, y*) = 0.0kPa, ∀(*x, y*) ∈ Γ_*i*_, and *p*(*x, y*) = 2.0kPa, ∀(*x, y*) ∈ Γ_*o*_, see Figure 5A;
- 2nd (ischemia): In a small region of the right-hand side of Γ_*o*_ pressure is reduced (to simulate a 30% stenosis), *p*(*x, y*) = 1.4kPa. The exact location is presented by the arrow in Figure 5B. In the rest of Γ_*o*_, pressure is set to its normal value, *p*(*x, y*) = 2.0kPa. See the circular color bar around the epicardial surfaces that presents pressure values at the epicardial surface in Figure 5B. At the endocardial surface *p*(*x, y*) = 0.0kPa, ∀(*x, y*) ∈ Γ_*i*_;
- 3rd (infarct): In a small region of the right-hand side of Γ_*o*_ pressure is reduced (to simulate a 80% stenosis), *p*(*x, y*) = 0.4kPa. The exact location is presented by the arrow in Figure 5C. In the rest of Γ_*o*_, pressure is set to its normal value, *p*(*x, y*) = 2.0kPa. See the circular color bar around the epicardial surfaces that presents pressure values at the epicardial surface in Figure 5C. At the endocardial surface *p*(*x, y*) = 0.0kPa, ∀(*x, y*) ∈ Γ_*i*_. In addition, this scenario also considers the presence of a fibrotic region near the endocardial surface and opposed to the simulated stenosis. This fibrotic region is called diseased, and its location and size are presented in Figure 5D.

The 3rd scenario has more particularities than just the pressure gradient. As mentioned before, the CA is confined to the interstitial space, and the measurements of this volume have several particularities (Al-Wakeel-Marquard et al., 2017). For the purpose of this work, we use λ = 0.25 (Arai, 2011) for the healthy and ischemic scenarios. In the case of an infarct region, many cells are dead and thus the fraction related to the interstitium is big, and we set λ = 1.0. In the same way, λ_*f*_, the fraction of fibrosis, is zero for the healthy and ischemic cases, whereas for infarct λ_*f*_ = 0.50. Finally, for the infarct scenario the wash out of CA is delayed, since it is trapped in the fibrotic network. Therefore, the rate of CA from fibrosis to the venous system is *k*_*f*_ = 0.0007 *s*^−1^, much slower than for the cases without fibrosis, where *k*_*e*_ = 0.003 *s*^−1^.

The total porosity ϕ was assumed to be constant and equal to 0.10 (Cookson et al., 2014). The Gaussian formula (Equation 11) was applied only at the epicardium, Γ_*o*_, with σ = 7.0 and *t*_*peak*_ = 22 s. The simulation time was set to 600 s, which is the average time used for the LE exam (Knowles et al., 2008; Arai, 2011). It is known that the exchange of oxygen, nutrients and CA from capillaries to the tissue is a phenomenon that involves different components of the biological system, such as the capillary, endothelium, interstitium, and parenchymal cells) (Bassingthwaighte et al., 1989). However, here we fix the endothelial permeability *P* of Equation 8 to *P* = 0.03 *s*^−1^, in the three scenarios. Nevertheless, section 4 presents a sensitivity analysis of this parameter. With respect to the 1D model, the parameter values were: $\vec{v}=1.1$ *mm* *s*^−1^ (velocity), $D_{out}=0.5\times 10^{-1}$ *mm*^2^ *s*^−1^ (diffusion coefficient), *k* = 0.015 *s*^−1^ (elimination of contrast in the recirculation). Table 1 presents a summary of the parameter values for each scenario.

**Table 1:** Parameters used in each scenario.

**Parameter (unity)**	**Healthy/Ischemic**	**Infarction**
*k*_*e*_ (*s*^−1^)	0.002	0.0009
*P*(*s*^−1^)	0.03	0.025
$k_{f}\left(s^{-1}\right)$	0	0.0007
$k_{ef}\left(s^{-1}\right)$	0	1.0
ϕ	0.10	0.10
λ	0.25	1.0
λ_*f*_	0	0.5
*D*(*mm*^2^*s*^−1^)	10^−3^	10^−7^
*K*(*mm*^2^*kPa*^−1^*s*^−1^)	*K*_1_ = 1.5; *K*_2_ = 0.75	*K*_1_ = 1.5; *K*_2_ = 0.75
σ	7.0	7.0
*t*_*peak*_(*s*)	25	25
$v_{out}\left(mm.s^{-1}\right)$	1.0	1.0
$D_{out}\left(mm^{2}s^{-1}\right)$	0.05	0.05
*k*(*s*^−1^)	0.02	0.02

### 3.2. Anisotropy and Heterogeneity of the Intravascular Domain

The subendocardial permeability is, in average, two times higher than the subepicardial one (Smith et al., 2014). In addition, the preferential direction of permeability, in the short-axis, is parallel to the epicardial and endocardial surfaces (Smith et al., 2014). Therefore, in order to take these features into account, a permeability tensor **K**(*x, y*) was generated ∀(*x, y*) ∈ Ω_*i*_. First we generate a transmural gradient of permeability by solving the Laplacian equation, Δ*w* = 0 ∈ Γ_*i*_, with *w* = 0 ∈ Γ_*o*_ and *w* = 1 ∈ Γ_*i*_, i.e., using Dirichlet boundary condition at the epicardial and endocardial boundaries, respectively. This gives us values between 0 and 1 for each node in the simulated domain. Next, we compute the pair of vectors {**e_*l*_**, **e_*t*_**}, where **e_*l*_** is the first and **e_*t*_** the second preferential direction of permeability by making **e_*t*_** = ∇*w*, and ensuring that **e_*l*_** is perpendicular to **e_*t*_**. With these two vectors calculated for each point (*x, y*) we can write the permeability tensor **K**(*x, y*) as follows:

(19) $$K(x,y)=K_{l}(x,y)I+(K_{t}(x,y)-K_{l}(x,y))e_{l}(x,y)e_{l}{\left(x,y\right)}^{t},$$

where *K*_*l*_ is the permeability value along *e*_*l*_ and *K*_*t*_ is the permeability along *e*_*t*_ (transversal to *e*_*l*_). These values can be calculated using the weight *w*:

(20)  Kl(x,y) = K1(1 − w(x,y)) + 2.0 K1 w(x,y),

(21) Kt(x,y) = K2(1 − w(x,y)) + 2.0 K2 w(x,y),

where *K*_1_ and *K*_2_ are the permeabilities in outer boundary, with *K*_1_ approximately 10.8 times higher than *K*_2_ (Smith et al., 2014); and the multiplication by 2.0 in the equation reflects that the subendocardial permeability is, in average, two times higher than the subepicardial one (Smith et al., 2014).

Table 1 presents a summary of the parameters used for each considered scenario in this work.

### 3.3. Computing

The simulator is an in-house code implemented in C. Simulations were executed on an Intel(R) Core(TM) i7 3 GHz 8 Gb. Each simulation of the late enhancement protocol (10 min of perfusion) takes approximately 708 s. This first version of the code was developed as a sequential one, i.e., parallel programming was not used, but it is planned for future works.

## 4. Results

Section 4.1 presents the pressure profile obtained from the Darcy approach (Equation 2). In the sequence, we present qualitative results of the CA perfusion on a short axis. Both, first pass and late enhancement are considered for the three different scenarios (healthy, ischemic, and infarction). In section 4.2 we present quantitative results of the model, and compare the signal intensities of the CA in the first pass and in the late enhancement exams to clinical data from the literature (Knowles et al., 2008; Daly and Kwong, 2013).

### 4.1. Spatio-Temporal Dynamics of CA Perfusion in the Heart

**Figure 6:**
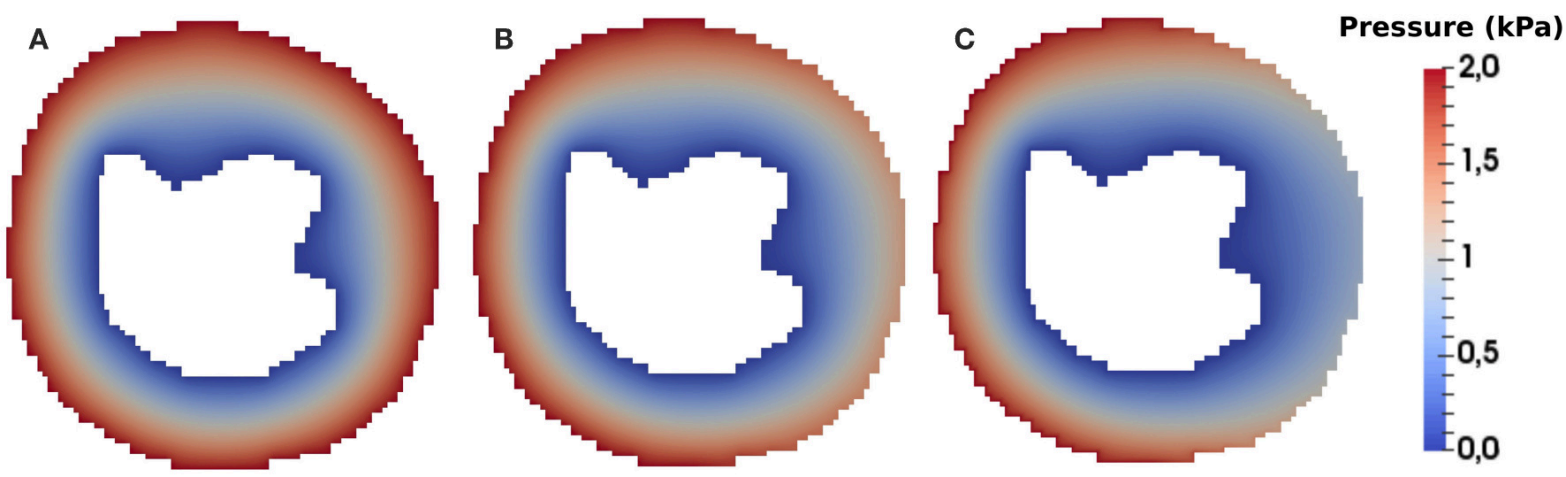
Pressure field of the **(A)** healthy **(B)** ischemic and **(C)** infarction scenario (in *kPa*). It is possible to observe that the pressure is higher near the epicardium area and lower near endocardium, as expected. The Dirichlet boundary condition mentioned in section 3.1 generates a pressure gradient. Using the Darcy's law we obtain a velocity field that is used in the advective term of Equation 6.

Figure 6 presents the numerical solutions obtained for the pressure distribution for the three considered scenarios: (a) healthy situation; (b) for the case where a stenosis of 30% is simulated; and (c) for a stenosis of 80%. In the real phenomenon, the pressure profile changes during the cardiac cycle. As mentioned before, we use the same hypothesis as the clinicians and evaluate the dynamics of perfusion at the same instant of each cardiac cycle, i.e., heart contraction is not taken into account. The calculated pressure and velocity will be used in the transport equation (Equation 6). From the figure we observe that the stenosis decreases the trasmural gradient of pressure.

**Figure 7:**
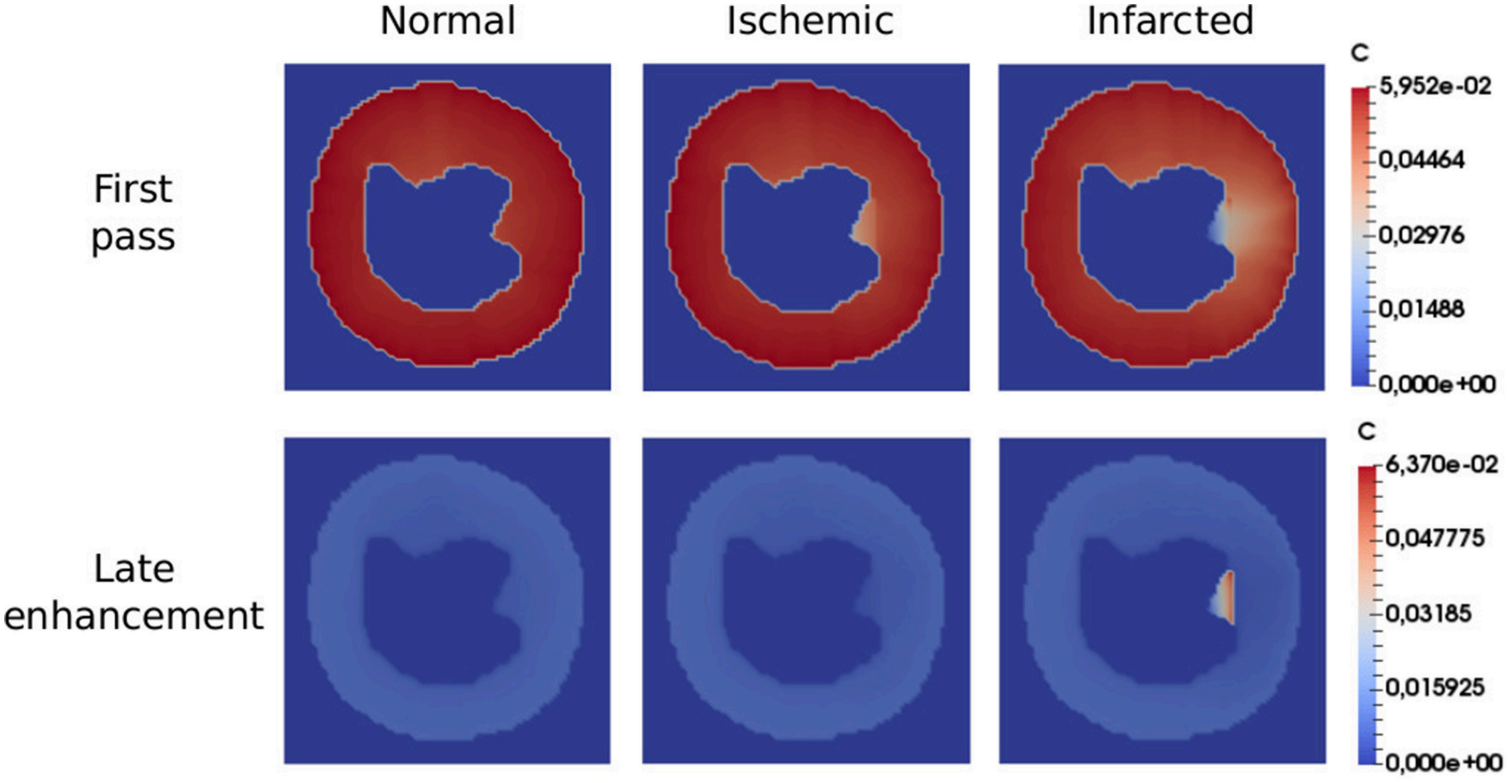
Qualitative images in the end of first pass (50 s) and late enhancement (600 s) exams. In the first scenario (healthy), the CA is homogeneously distributed, while in the second (ischemia), the diseased region is revealed by low localized contrast values. At the time of late-enhancement, the CA gets stuck in the infarction region as reflected by the high localized contrast values. All the simulations reproduced well the characteristics seen by the clinicians in the considered scenarios.

Figure 7 shows the final result, in terms of qualitative images of the myocardial short axis for the three simulated scenarios. For each scenario, we shown the concentration in the end of both first pass and late enhancement exams. The simulation domain was adapted from a real short axis MRI image (Koch et al., 2011). The model reproduces very well the qualitative behavior, for both first pass and late enhancement exams. During first pass, in a healthy heart the CA is homogeneously distributed, whereas in the cases of ischemia and infarction the affected region is revealed by low localized contrast values. At the time of late-enhancement, the CA attaches to fibrosis in the infarct region. This is reflected by the high localized contrast values at late-enhancement.

### 4.2. Signal Intensity of Contrast Agent

The variable of interest in this section is the signal intensity of the contrast agent in specific regions of interest (ROI). To compare the CA dynamics of the healthy and pathological cases, two regions of interest in the myocardium were marked as presented in Figure 5D: one captures the injured region and the other is on a remote healthy region. Each value of the time series correspond to the sum of the CA concentration in one specific ROI at a specific time.

Since we are setting a reduced pressure on the right-hand side of the epicardial surface to simulate a stenosis, we placed the diseased ROI as a subendocardial region opposite to the stenosis point. The remote ROI was placed on the subendocardial left-hand side of the short axis (see Figure 5D). These are common choices of ROIs to compare a diseased versus a healthy region (Hsu et al., 2006; Schuster et al., 2015). In our simulations, the remote ROI has a total area of 70 *mm*^2^, while the diseased ROI has 55 *mm*^2^. These sizes and locations of ROIs are commonly reported in the literature (Hsu et al., 2012; Pop et al., 2013; Schuster et al., 2015). For the third scenario that simulates an infarct, a fibrotic region was modeled at the same location of the diseased ROI.

Section 4.2.1 presents the results for the ischemic scenario. Section 4.2.2 presents the results of the infarction. Is important to remark that the fibrosis mentioned before was placed only in the *diseased* region, with a particular volume fraction, as discussed in section 2.1.

#### 4.2.1. First Pass

**Figure 8:**
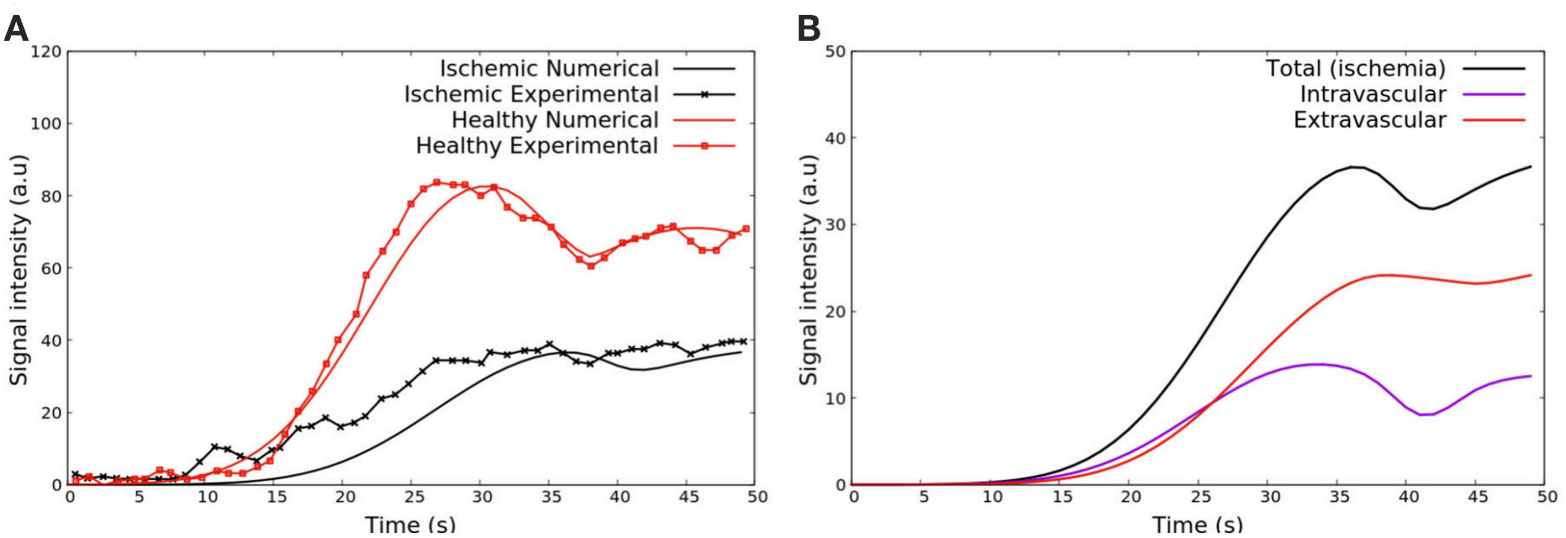
**(A)** Comparison between SI of the CA during time observed in Daly and Kwong (2013) and the numerical results of this work in some ischemic area (red) and remote and healthy area (blue). The oscillatory decay is due to the Gd-DTPA recirculation. **(B)** The contribution of intra- and extravascular SI of the CA in the ischemic area: (purple) total, (red) intravascular, and (black) extravascular concentrations vs. time.

Figure 8A shows the results obtained with respect to the intensity of CA signal during a first pass MRI perfusion exam in the two considered regions of interest. These results are in agreement with those found in the literature (Nagel et al., 2003; Jenson et al., 2013; Wang et al., 2015; Bakir et al., 2016). In particular, the experimental curves presented in Figure 8A are taken from Daly and Kwong (2013), where the first pass of healthy region is compared to a ischemic one. It is possible to observe the *second pass* of the CA, i.e., the remaining amount of it that was not eliminated by the kidneys and recirculates. Figure 8B presents how each domain, intra and extravascular, contributes to the total SI of CA over time for the case of ischemia. These results are also in agreement with the literature (Bogaert et al., 2005).

#### 4.2.2. Late Enhancement

**Figure 9:**
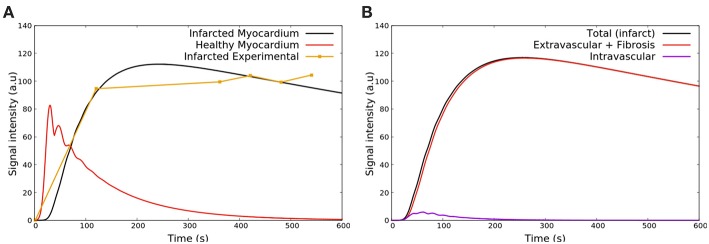
**(A)** SI of the contrast agent in scenarios remote/healthy (red) and infarction (black). The yellow curve presents the clinical data taken from Knowles et al. (2008). The CA remains in the infarct area because it attaches to fibrosis, and thus takes longer to be washed out. **(B)** Contribution of both intra and extravascular spaces in the CA dynamics in the infarct region. The intravascular curve (purple) shows that the CA in this domain is quickly washed out, while the CA remains in the extravascular space (red curve) after 2 min.

After the first pass detects a region with poor perfusion, late-enhancement is performed to study myocardial viability, i.e., to differentiate between ischemia, but yet viable tissue, or infarct. This is possible because fibrosis is present in chronic infarct and the CA is trapped in the fibrotic network, taking longer to be washed out of the myocardium. If the perfusion problem is not so severe, like under ischemia, the CA is washed out at a similar rate as a remote/healthy region. The results shown in Figure 9 are in agreement with the late enhancement dynamics (Arai, 2011) and reproduces well the clinical data taken from Knowles et al. (2008). Figure 9 presents the contribution of both intra- and extravascular spaces in the CA dynamics in the infarct region. The intravascular curve shows that the CA in this domain is quickly washed out if compared to the CA in the extravascular space.

## 5. Discussion

As we present a new model for cardiac perfusion with focus on the application of contrast-enhanced MRI, this section begins by presenting sensitivity tests of the proposed models. This will contribute to the discussion regarding the importance of our main hypothesis, the positive outcomes of our model as well as its limitations.

### 5.1. Sensitivity of the Parameters

This section is divided in three parts. Section 5.1.1 shows the importance of the recirculation coupling in the model (Equation 18). In section 5.1.2, an effort to reproduce clinical data of LE for the infarction scenario without taking into account the domain of fibrosis is presented. In section 5.1.3, we demonstrate that the coupling of the model with a fibrotic domain and the inclusion of a sortion term provide a better alternative to model the experimental and clinical data.

#### 5.1.1. Recirculation

**Figure 10:**
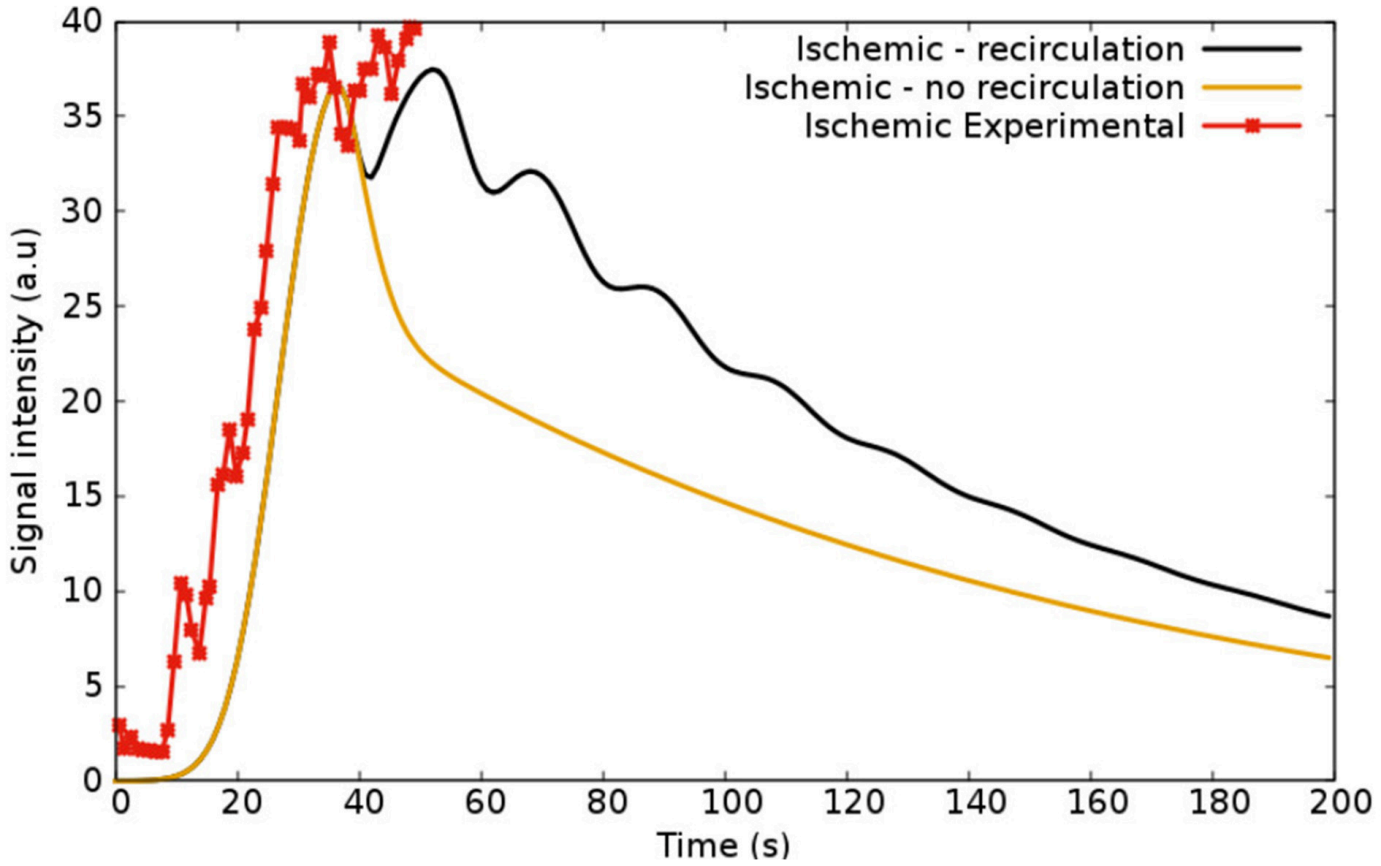
Simulations regarding ischemic scenarios: the first (black), shows the model coupled with the recirculation part (Equation 18) and the second (yellow), without it. The red curve shows the experimental data from Daly and Kwong (2013).

Figure 10 is presented to reinforce the idea that the recirculation coupling is important to obtain results close to those obtained in clinical exams. The red curve is the clinical information from Daly and Kwong (2013) (same as in Figure 8A), and due to the lack of data, it stops after 50 s. The figure also shows the dynamics of the CA with the recirculation model (black, same as in Figure 8A) and without it (yellow) for 200 s of perfusion. As presented, if one does not take into account the recirculation model and its parameters (${\vec{v}}_{out}$, *D*_*out*_ and *k* from Equation 18), the oscillatory decay observed in the clinical and experimental data is not captured.

The whole process of recirculation is quite complex: the kidneys, for instance, play an important role in the physiological process of elimination of CA; the constant flux velocity assumed by the model is in fact a simplification, since the flux varies and depends on the location if the body, the structure of the vessels, viscosity, and many other features. Therefore, other variables and parameters could be added in this part of the model. However, it is not in the scope of this work to go deep into this topic. The solution presented here is taking into account the blood cycle in the whole human body as a single-phase flow in a simple 1D domain, which provides good qualitative agreement with experimental data. For future works, it would be interesting to study this phenomenon and look for other solutions.

#### 5.1.2. Modeling Perfusion Without a Mathematical Description of Fibrosis

As mentioned before, without taking into account (Equation 9), that describes fibrosis, the model captures well the scenario of ischemia and is able to reproduce clinical data (Daly and Kwong, 2013). However, here we show that without the mathematical description of fibrosis the model can not reproduce the dynamics of infaction, mainly those found in the time-scale of the late-enhancement exam.

**Figure 11:**
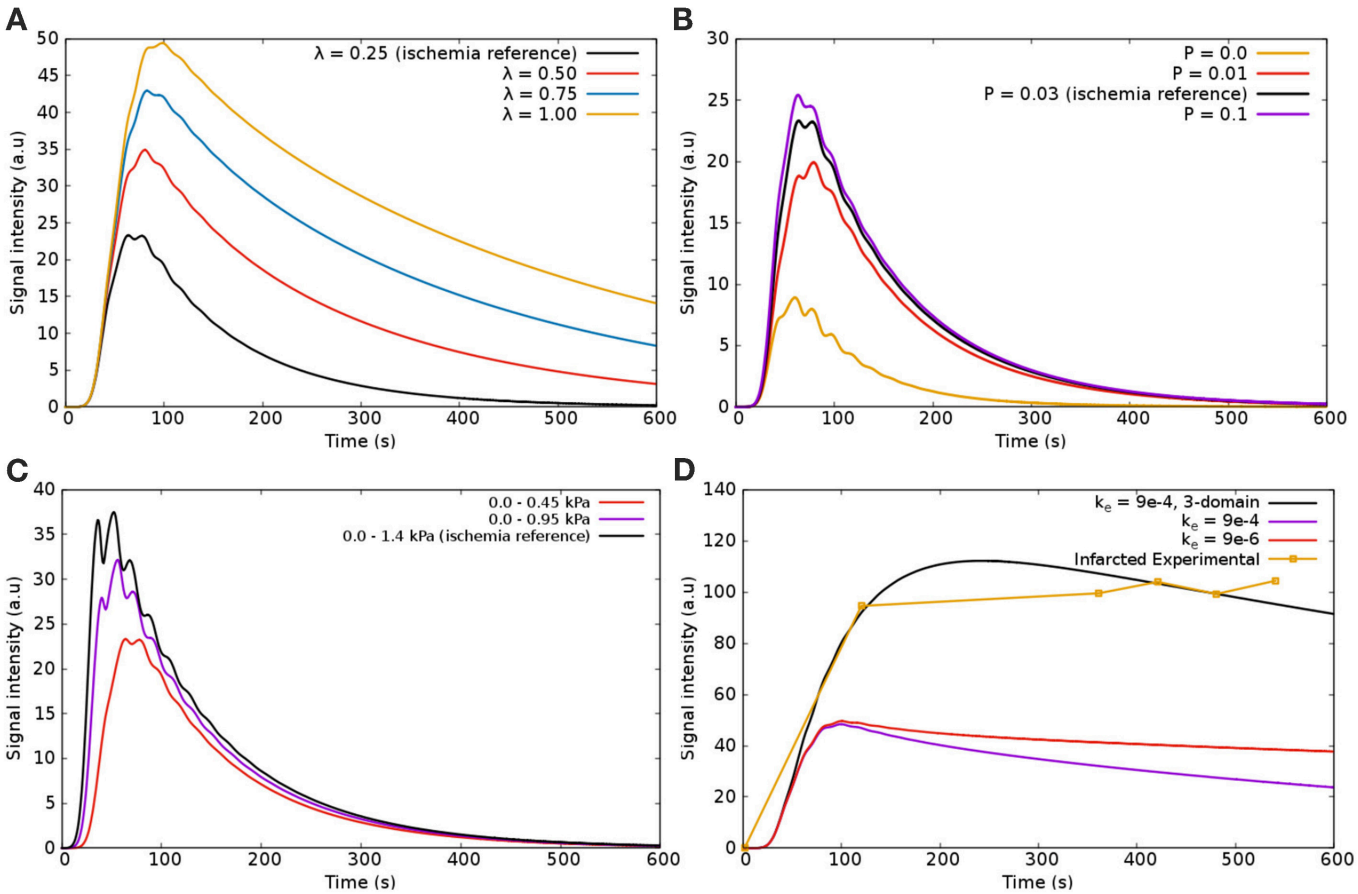
Sensitivity analysis of the parameters using the model two domains: **(A)** λ—the fraction of extravascular media that corresponds to the interstitium; **(B)** *P*—the rate of CA that goes from intravascular to extravascular space; **(C)** Pressure difference between epicardium and endocardium in the infarcted region; **(D)** *k*_*e*_—the rate of CA going from extravascular media to the venous domain (a term of decay). When varying one parameter, all the others are fixed according to the infarction column in Table 1.

Figure 11 presents the sensitivity analysis of the model without the coupling to Equation (9). In other words, it shows an attempt of reproduce the clinical data of infarction from Knowles et al. (2008) using just a part of the proposed mathematical model. All curves in Figure 11 refer to the dynamics of the CA in the diseased region according to Figure 5D. The parameters were only varied is this ROI, and kept constant in the other regions. In addition, when varying one parameter, all the others are fixed according to the infarction column in Table 1.

Figure 11A presents the sensitivity of λ (fraction of interstitium in the extravascular domain). For high values of λ there is more space for the CA. Thus, more CA is hold in the interstitial domain: time-to-peak, peak value and value at 600 s increase. These are all in the correct direction to reproduce the data in Figure 11D (black and yellow). However, even setting λ = 1, i.e., to its maximum value, the values of Figure 11A are far from those of Figure 11D (black and yellow). Figure 11B shows the sensitivity of *P*, which models the rate of CA exchange between intra and extravascular domains. For instance, *P* = 0 *s*^−1^ indicates that there is no contrast agent flowing from one domain to the other, which means that it is confined to the intravascular domain. Hence, in this particular case, the CA is quickly washed out. Increasing *P*, the CA goes faster to the extravascular domain, hence it takes longer to be washed out to the venous system, but it is still completely washed out at 600 s. Figure 11C shows how the pressure gradient, which is related to the severity of coronary stenosis, influences the signal intensity of the CA in the diseased region. For example, in the case of an ischemia (this one is related with Figure 5B), the obstruction of 30% leads to a pressure gradient of up to 1.45 *kPa* in the right portion of the myocardium. In the worst scenario shown here, an obstruction of 80% leads to a pressure gradient of up to 0.45 *kPa* between epi- and endo-cardium (related with Figure 5C). As expected, the pressure gradient is directly proportional to the rate with which the CA reaches the diseased area. In Figure 11D, two different values for *k*_*e*_ (transfer of CA from the extravascular media to the venous system) are used. As expected, decreasing the value by two order of magnitude, the CA takes longer to be washed out. However, once gain, the curves obtained are far from the goal 11D (black and yellow).

In summary, using the formulation with only two domains (intra- and extra-vascular), it was not possible to reproduce the observed scenario of CA dynamics for a case of infarction. However, in Cookson et al. (2014), a formulation with two domains was used and some results were similar to those observed for infarction. To achieve this, however, the authors had to change the permeability of the media (**K** in Equation 3) in specific regions. Their purpose was to evaluate the CA dynamics in different scenarios, without specifying any pathology. For the cases of healthy, ischemic or infarct myocardium, the three scenarios treated here, the changes used in the permeability **K** would be difficult to justify. Thus, for the simulation of an infarction it seems to be important to model fibrosis, as we propose in our new formulation.

#### 5.1.3. Modeling Perfusion With a Mathematical Description of Fibrosis

**Figure 12:**
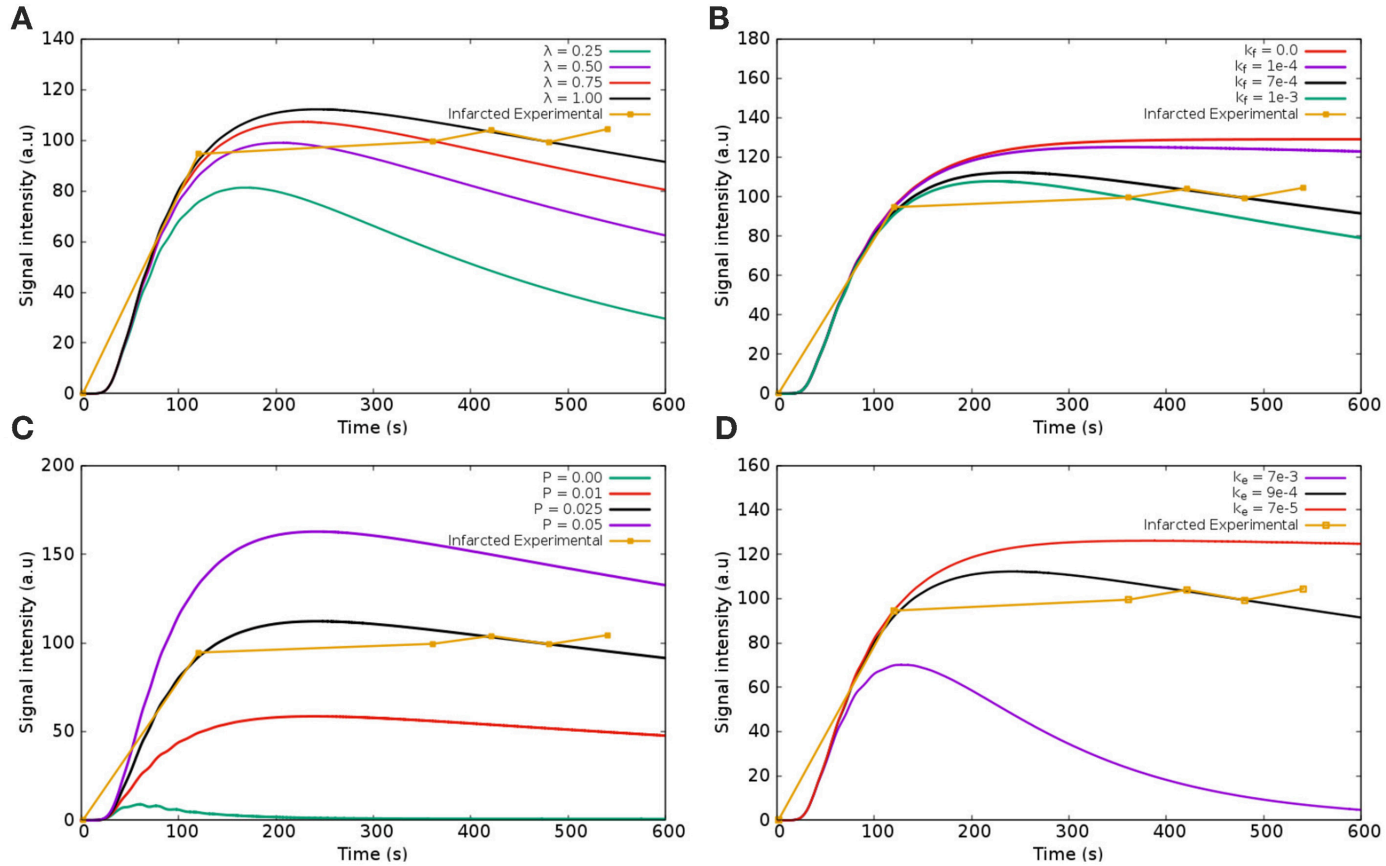
Sensitivity analysis of the parameters in the infarcted scenario using the model with three domains: **(A)** λ—the fraction of interstitium in the infarcted area; **(B)** *k*_*f*_—the exchange rate of CA going from interstitium to the venous system; **(C)** λ_*f*_—the fraction of interstitium that has fibrosis (scars); **(D)** decay of CA from the fibrosis. When varying one parameter, all the others are fixed according to the infarction column in Table 1.

Figure 12 shows the dynamics of the CA in the infarct region taking into account the third domain and Equation 9. Figure 12A shows the dynamics of the CA for different values of λ (interstitial fraction of the extravascular media). For the healthy regions, its values is around 0.25 (Knowles et al., 2008; Arai, 2011). Once again, for all the tests, when varying one parameter, all the others are fixed according to the infarction column in Table 1. As in Figure 11A, for high values of λ there is more space for the CA. Thus, more CA is hold in the interstitial domain. However, using this model, time-to-peak, peak value, and value at 600 s are all much more sensitive to λ. And therefore, few adjustments of the parameter are enough to fit different experimental values. Figure 12B presents the sensitivity of *k*_*f*_, the rate in which the CA goes from fibrosis to the venous system. The higher its value, the faster the CA is washed out of the myocardium. If *k*_*f*_ = 0.0 *s*^−1^, no CA is leaving the fibrotic region, hence a plateau appears. The best value that reproduces the clinical data is $k_{f}=7\times 10^{-4}$ *s*^−1^. Figure 12C presents the sensitivity of *P*. The value that best fitted the clinical data of infarction is *P* = 0.025 *s*^−1^. Once again, if *P* = 0.0 *s*^−1^, there is no communication between intra and extravascular domains, hence the CA is quickly washed out. Figure 12D shows results for different values of *k*_*e*_ (decay rate of CA). It reaches saturation at 7 × 10^−5^ *s*^−1^. In summary, the presented formulation that models a third domain for fibrosis has proved to be a good alternative for the simulation of the dynamics of CA in the case of infarction. In addition, the curves generated with this model were more sensitive to many of its parameters. Therefore, a few adjustments of some parameters are enough to fit different experimental values.

### 5.2. Limitations and Future Works

For low doses of contrast agents, there is an approximately linear relationship between the SI of the CA and its concentration (Donahue et al., 1997). For cardiovascular imaging, the dosage is not low enough for this linearity assumption to apply (Gerber et al., 2008). To overcome this issue, several researchers have presented different solutions (Cernicanu and Axel, 2006; Engblom et al., 2017; Kellman et al., 2017). In this work, the presented models focus on the dynamics of CA concentration. In future works, we will adopt the aforementioned solutions to convert CA concentration to SI and vice-versa.

As mentioned before, in the series of images provided by the contrast enhanced MRI perfusion, the clinicians take the same image, or phase, at different cardiac cycles to analyze the perfusion of the CA. Doing so, the short axis images of the left ventricle have the same shape over time. Hence it is reasonable to assume a static configuration of pressure. However, this assumption, which does not take into account poroelastic effects, has its shortcomings. To improve this model, a general mathematical model that couples porous flows and hyperelastic formulation was developed in Chapelle and Moireau (2014). Furthermore, the same authors used a similar formulation in order to evaluate myocardial perfusion (Chapelle et al., 2010). In future works, the model presented here will be extended in this direction, in order to obtain more reliable results and to evaluate the myocardium at different scenarios and for different pathologies.

It is important to mention that the pressure and flow distributions in the intravascular tree are more complex than those obtained by our model. The fine and complex structure with its connectivity, branches and varying radius are important features that are not present in our continuum porous media model. Therefore, in the near future we will replace our continuum approach for the intravascular domain by a detailed arterial tree model. In order to generate arterial trees, we will adopt algorithms based on the CCO (Constrained Constructive Optimization) method (Karch et al., 1999; Blanco et al., 2013; Brito et al., 2017; Meneses et al., 2017). This will allow us to model stenosis in a more precise way, by directly altering the radius of a particular branch of the coronary arteries.

We have chosen to use a segmentation of the short axis that includes the papillary muscles. Indeed, recent papers show that these muscles have impact on the analysis of MR images (Gommans et al., 2016). However, this controversy is out of the scope of our work. The segmentation choice was arbitrary and our model and implementation is general enough to use any domain, short-axis, long-axis, with or without papillary muscles. The same applies for the location and shapes of the modeled infarct region.

A very important feature here presented is the simulation of fibrosis. To reproduce late-enhancement images for the case of infaction it was fundamental to couple the third domain for fibrosis to the two-domain model (intra- and extra-vascular domains). However, this brings more parameters to be adjusted. In future works, we plan to study how the numbers of parameters of the models affect the correct description of patient specific perfusion data.

A similar model to the two-domain model (intra- and extra-vascular domains) presented here has been successfully used in the quantification of myocardial perfusion with cardiac MRI (Engblom et al., 2017; Kellman et al., 2017; Brown et al., 2018). It is a simplification of the model presented in Bassingthwaighte et al. (1989) and was called the BTEX model. This simplified model was shown to be very useful to speed up the inverse problem of parameter estimation to reproduce patient-specific CA dynamics. Here, we will show the simplification steps needed to convert our two-domain model into the BTEX model. First, the pressure equation is not taken into account, and tissue perfusion is characterized by a single scalar parameter F which replaces our vector $\vec{v}=-\text{K}\nabla p$ in Equation (6). Another simplification of BTEX is that it is used as a set of independent 1D two-domain models. The perfusion F is independently estimated for each MRI pixel via a 1D model. This approach allows a great simplification for the problem of parameter estimation. In this work, we have presented 2D models that can be easily extended to full 3D models of the heart. Finally, a very important feature here presented was the simulation of fibrosis. To reproduce late-enhancement images for the case of infarction, it was fundamental to couple the third domain for fibrosis to the two-domain model. In summary, the new models proposed in this work have some potential advantages for the process of cardiac perfusion quantification. However, the models have more equations and parameters than the simplified models that are currently used. As a consequence, parameter estimation via the solution of an associated inverse problem will be a difficult task. One solution is to use additional non-invasive information to adjust the model, such as T1 and T2 mapping from MRI exams and FFR (pressure and flux) estimations from CT exams. As future work, we plan to study how these larger number of parameters and equations may affect the correct estimation of patient-specific cardiac perfusion by performing a clinical study similar to those presented in Engblom et al. (2017), Kellman et al. (2017), and Brown et al. (2018).

### 5.3. Conclusion

We presented a mathematical model that describes the blood and contrast perfusion in a short axis cut of the left ventricle of the heart. The simulation results are in agreement with the observed phenomena, specifically with the MRI images obtained by the non-invasive first pass and late enhancement exams and with clinical data on the time dependence of the contrast agent (CA) in different situations. This model takes into account the flow of Gadolinium-based MRI CA (Gd-DTPA) in the arterial vascular domain, its leakage to the interstitial domain and its retention in fibrotic tissue (when simulating a scenario of infarction). Having in mind the substantial information that quantitative methods can provide for the physicians, the mathematical model proposed here and its future extensions can be considered as a first step to develop better tools to understand the complexity of the spatio-temporal CA dynamics and the relation between clinical data acquired via MRI and the underlying cardiac perfusion.

## Author Contributions

JA and RdS conceived the mathematical model. JA implemented the code and performed the simulations. JA and RdQ developed the numerical approximations. MB provided clinical data to validate the model. All authors evaluated the results and reviewed the manuscript.

### Conflict of Interest Statement

The authors declare that the research was conducted in the absence of any commercial or financial relationships that could be construed as a potential conflict of interest.
